# Reduced Descending Itch Inhibition in Peripheral Neuropathy Patients With Chronic Pruritus

**DOI:** 10.1002/ejp.70190

**Published:** 2026-01-03

**Authors:** Jonas Eck, Stephan Bigalke, Martin Schmelz, Daniela Constanze Rosenberger, Bruno Pradier, Frank Rutsch, Claudia Sommer, Frank Birklein, Daniel Segelcke, Esther Pogatzki‐Zahn

**Affiliations:** ^1^ Department of Anaesthesiology Intensive Care and Pain Medicine University Hospital Münster Münster Germany; ^2^ Department of Orthopedics and Trauma Surgery Freiburg University Hospital Freiburg Germany; ^3^ Department of Anaesthesiology Intensive Care and Pain Medicine BG University Hospital Bochum Bochum Germany; ^4^ Department of Experimental Pain Research, Medical Faculty Mannheim University of Heidelberg Mannheim Germany; ^5^ Department of General Pediatrics Muenster University Children's Hospital Münster Germany; ^6^ Department of Neurology University Hospital Würzburg Würzburg Germany; ^7^ Department of Neurology University Medical Center of the Johannes Gutenberg University Mainz Mainz Germany

## Abstract

**Background:**

Peripheral neuropathies (PNP) cause a wide range of symptoms of which pruritus and pain are a major burden to patients. Clarifying their mechanisms will facilitate the adequate symptom management. In contrast to neuropathic pain, neuropathic pruritus has long been understudied. We investigate inhibitory pruritus mechanisms in patients with PNP.

**Methods:**

A total of 39 participants (median age: 57 years [27–81], 25♀/14♂), including 16 patients with PNP and pruritus (PNP_PRU_) and 8 with PNP but without pruritus (PNP_NO‐PRU_), and 15 controls, were enrolled and phenotyped using dynamic and static quantitative sensory testing, determination of intradermal nerve fibre density, and conditioned pain (CPM) and itch (CIM) modulation. For CIM, an electrically induced itch test stimulus (TS) was applied with an individual itch intensity of > 30 NRS (0–100). A cold‐water bath (contralateral side) served as the conditioning stimulus (CS). Itch, pain intensity and the desire to scratch upon TS were rated before, during and after the CS was applied.

**Results:**

Using our novel CIM protocol, well‐controlled and stable itch was electrically induced in controls and in both PNP groups, regardless of whether or not pruritus was a symptom. Concomitant painful cold stimuli reduced electrically induced itch in all three groups to a similar degree. However, PNP_PRU_ patients had significantly shorter itch inhibition (CIM effects) compared to controls and PNP_NO‐PRU_, whereas CPM effects were not different between groups.

**Conclusions:**

The results show reduced endogenous itch inhibition in patients with chronic itch, which represents a possible mechanism of chronic pruritus in PNP.

**Significance Statement:**

Reduced itch inhibition is associated with the symptom of chronic pruritus in PNP patients, suggesting that reduced descending itch inhibition may facilitate chronic itch in peripheral neuropathy.

## 1 Introduction

Peripheral neuropathies (PNP) may have very different causes. Chronic neuropathic pain, neuropathic pruritus and various other sensory symptoms are commonly observed in PNP patients, causing significant personal and economic burden (Baka et al. 2024). Although pain has been extensively studied in PNP patients, chronic neuropathic pruritus (nPru) as a form of chronic itch remains poorly understood (Steinhoff et al. 2019). Despite a significant impairment of quality of life, there are no approved treatments for nPru (Kwatra et al. 2023). The neglect of nPru in medical research and practice underscores the need for increased awareness and improved diagnostic and therapeutic strategies.

Currently, there is compelling evidence from human studies showing that nPru may arise from the activation and sensitisation of specific pruriceptors or subsets of nociceptors (LaMotte et al. 2014; Pogatzki‐Zahn et al. 2020; Schmelz 2019), such as C‐fibres expressing Mas‐related G‐protein coupled receptors (MrgprA3, MrgprC11), histamine receptors (H1R, H4R) and TRPV1 and TRPA1 channels (Han et al. 2013; Misery et al. 2023). While the activation of a larger number of afferent fibres leads to pain, pruritus is inhibited at the same time (Hu et al. 2021). The theory of spatial contrast puts forth an alternative explanation, suggesting that pruritus is caused by the divergent pattern of active and inactive nociceptors from the same skin site (Namer and Reeh 2013; Steinhoff et al. 2019). However, in humans, this theory was refuted recently (Klein et al. 2021), who demonstrated that the spatial contrast hypothesis does not hold in human pruriceptive coding. Their work demonstrated marked species differences in nociceptor function and provided important insights into why findings from animal models cannot always be directly translated to humans. This underscores the need for conducting experimental studies in humans to attain a thorough and more accurate understanding of the mechanism underlying chronic pruritus in patients (Klein et al. 2021). Furthermore, past research has showed that impaired endogenous sensory modulation and sensitisation play a role in the progression or persistence of chronic pain and itch in individuals with chronic pruritic conditions (e.g., atopic dermatitis or psoriasis) (Ikoma et al. 2003; Mochizuki et al. 2003; van Laarhoven et al. 2007).

We recently showed that endogenous pain‐related inhibition, assessed via a conditioned pain modulation (CPM) paradigm, is markedly reduced in patients with chronic pruritus (Pogatzki‐Zahn et al. 2020). The experimental CPM paradigm has been extensively evaluated in prior studies involving patients with different chronic pain entities, consistently revealing a diminished endogenous inhibition in some but not all studies (Ossipov et al. 2014). These data suggest that dysfunction of descending pain inhibition occurs in a subset of patients and disease entities with chronic pain. While a causal link remains to be proven, the reduced inhibition might contribute to persistent chronic pain under certain conditions (Büchel 2022; Kwon et al. 2014).

It is still unclear whether descending inhibitory systems may also control itch perception and have a potential role in patients with chronic pruritus. In accordance with the CPM paradigm, numerous experimental studies have employed pruritic stimuli as the test stimulus (TS) and pain as the conditioning stimulus (CS), thereby establishing the paradigm known as conditioned itch modulation (CIM) that confirmed itch reduction upon painful, but not pruritic conditioning stimuli (Andersen et al. 2017; Granot et al. 2019; van Laarhoven et al. 2010). While most studies with CIM paradigms include healthy volunteers, there is only one study with patients (van Laarhoven et al. 2016). Consequently, the exact role of inhibitory mechanisms, specifically those that modulate chronic pruritus in patients, remains largely unresolved.

Here, we developed a novel, stable CIM paradigm using well‐controlled electrically induced itch and examined if itch inhibition by painful stimuli differs between PNP patients with and without chronic pruritus. We hypothesised that descending itch inhibition is reduced in the PNP patients with chronic pruritus and that the PNP patients without chronic pruritus (with and without pain) had a similar descending itch inhibition to the healthy controls.

## 2 Methods

In compliance with the latest version of the Declaration of Helsinki, this study was conducted and received approval from the ethics committee of the Faculty of Medicine, University of Münster, Germany (registration no: 2017‐576‐f‐S).

### 2.1 Study Design

**FIGURE 1:**
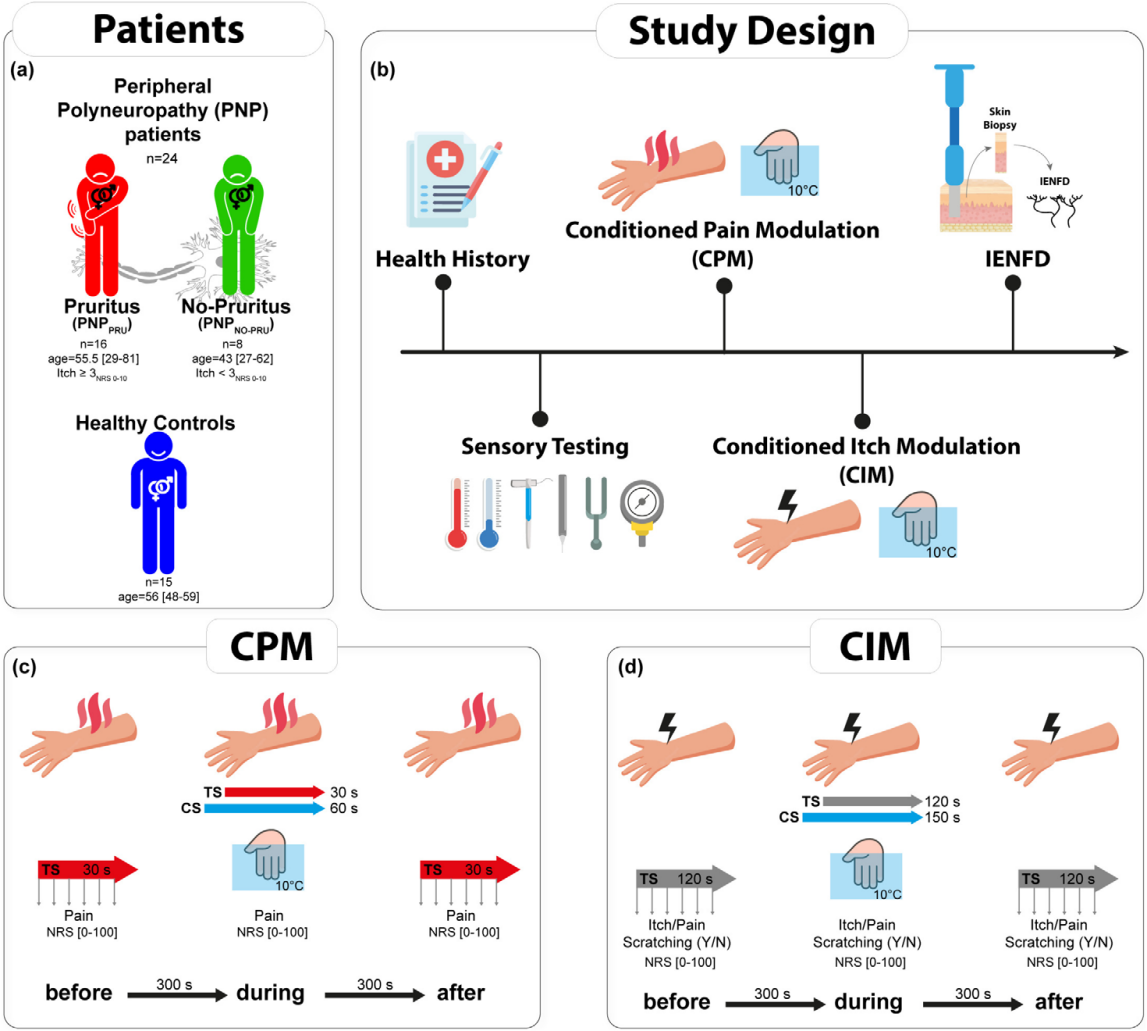
Groups and testing protocol schematic. (a) Participant groups, numbers and age (mean [range]). Healthy controls had no diagnosed peripheral neuropathies (PNP). PNP patients were divided depending on chronic pruritus as a symptom. PNP patients with a lower (< 3) average itch intensity were assigned to the PNP_NO‐PRU_ group. (b) Study design. (c) Conditioned pain modulation (CPM) protocol. (d) Conditioned itch modulation (CIM) protocol. Electrical TS was applied ‘before’, ‘during’ and ‘after’ the CS for 120 s each. Itch intensity, pain intensity and the desire to scratch were assessed in 20 s intervals during the test runs. For the test run ‘during’, the contralateral hand was immersed in 10°C cold water as a CS starting 30 s before the TS started. A break of 5 min was taken in between the test runs. CS, conditioned stimulus; N, no; NRS, Numeric Rating Scale; TS, test stimulus; Y, yes.

All patients and healthy controls were recruited for a multicentre trial published recently (Baka et al. 2024). For the current study, patients included in Muenster received additional assessments which are included in the present manuscript. Inclusion criteria were an age between 18 and 81 years, diagnosis of PNP of any aetiology and skin type I‐IV (Fitzpatrick 1988). Exclusion criteria were the use of antipruritic medication, infection at the examination area and cases in which the electrical stimulation did not induce an itch intensity of 30 or more (on a Numeric Rating Scale (NRS), 0–100) in a test run prior to the CIM protocol. Itch intensity was rated on a NRS from 0 to 100, where 0 represented ‘no itch at all’ and 100 indicated ‘the worst itch imaginable’. Healthy individuals were recruited through advertisements to form a control group. This group did not include participants who demonstrated any of the subsequent conditions: signs or symptoms of PNP, chronic or current pain or pruritus, psychiatric or neurological disorders, malignant conditions, dermatological diseases or any factor that could potentially lead to PNP. This encompasses conditions such as diabetes mellitus, abnormal glucose tolerance, harmful alcohol consumption, current or previous chemotherapy or a positive family history of PNP (Figure 1a). In the control group, pre‐existing conditions included arterial hypertension (5 of 15 individuals), hypothyroidism (3/15), bronchial asthma (2/15) and osteoarthritis (1/15). None of the subjects reported regular use of analgesic or antipruritic medication. All participants were instructed about testing procedures and associated risks at a separate time point before testing. All participants signed informed consent and were instructed that they could refuse participation at any point.

Demographic data, general medical and drug history and symptom duration were collected (for details, see (Baka et al. 2024)). Following the neurological examination, participants underwent several investigations: QST, CPM, CIM and skin biopsies to determine intraepidermal nerve fibre density (IENFD) (Figure 1b).

Based on the reported mean pain and pruritus intensity (11‐point NRS scale, 0–10) during the 4 weeks prior to inclusion (see Table 1 for mean and maximum values), PNP patients were divided into two subgroups: ‘pruritus (PNP_PRU_)’ and ‘no‐pruritus (PNP_NO‐PRU_)’. The subgroups were defined as follows: ‘PNP_PRU_’ with an NRS ≥ 3 for itch and ‘PNP_NO‐PRU_’ with an NRS < 3 for itch. Only patients with a pain intensity > 3 on the NRS were included. The decision to use an NRS cut‐off of 3 was based on the fact that it is widely used as an inclusion criterion for intervention trials (Birnie et al. 2012; Hoffman et al. 2010).

**TABLE 1:** Demographic and clinical characteristics of patient cohort.

	PNP_PRU_	PNP_NO‐PRU_	Controls
*n*	16	8	15
Age [years]	55.25 (29–81; SD 13.74)	44.00 (27–62; SD 12.74)	54.53 (48–59; SD 3.74)
Sex [f/m]	10/6	5/3	10/5
Mean reported average itch [NRS 0–10] in daily life	5.06 (3–10; SD 1.65)	0.50 (0–2; SD 0.76)	0.07 (0–1; SD 0.26)
Mean reported max. itch [NRS 0–10] in daily life	7.81 (4–10; SD 1.56)	1.00 (0–3; SD 1.41)	0.33 (0–5; SD 1.29)
Mean reported average pain [NRS 0–10] in daily life	3.88 (0–9; SD 2.5)	3.25 (0–5; SD 2.05)	0.00 (0–0; SD 0.00)
Mean reported max. pain [NRS 0–10] in daily life	6.81 (0–10; SD 3.76)	5.62 (0–10; SD 3.66)	0.00 (0–0; SD 0.00)

### 2.2 Sensory Testing

Quantitative Sensory Testing (QST) was performed unilaterally at two sites (distal lateral leg (test area) and the ipsilateral cheek (control area)), utilising the protocol of the German Research Network on Neuropathic Pain (DFNS) (Rolke et al. 2006), to evaluate sensory phenotypes for different stimulus modalities. The distal lateral leg was selected as the test area due to frequent involvement in length‐dependent neuropathies. The ipsilateral cheek served as a non‐affected control site for intra‐individual comparison. Measurements included thermal and mechanical detection and pain thresholds, paradoxical heat sensation, dynamic mechanical allodynia, wind‐up ratio and vibration detection threshold.

### 2.3 Conditioned Pain Modulation

The efficiency of endogenous pain inhibition was evaluated using the CPM paradigm, as previously described (Pud et al. 2009) (Figure 1c). Initially, heat stimulation that produced a pain intensity rating of approximately 60 on a 0–100 NRS (0 = ‘no pain felt’, 100 = ‘worst imaginable pain’) was identified using a 9 cm^2^ contact thermode (TSA II NeuroSensory Analyser, Medoc Ltd., Israel). Two sets of three 7‐s heat stimuli (45°C, 46°C and 47°C) were applied to both forearms (initially on the right forearm in ascending order, then on the left forearm in a randomised order). Participants were asked to rate the perceived pain intensity (NRS 0–100). The temperature causing an intensity of approximately 60 on the NRS was selected as the TS. If these temperatures resulted in pain ratings above 65 or below 55 (on the 0–100 NRS), additional series with lower (42°C and 43°C) or higher (48°C and 49°C) temperatures were applied to determine an appropriate stimulus intensity. Stimulation at 48°C–49°C, if required, was applied only in isolated cases and for brief 7‐s periods, with a maximum of three applications per arm. Skin was inspected after each stimulation, and no tissue damage, irritation, or adverse reactions were observed. If no suitable temperature could be identified within this range, the participant would have been excluded from the study. However, no such cases occurred. Next, participants received a 30‐s heat stimulation at the determined temperature of the TS and rated the pain intensity at 10, 20 and 30 s on an NRS (0–100) (TS_before_). After a 5‐min interval, participants immersed their contralateral hand in a 10°C cold water bath for 60 s and rated the pain intensity at 30 and 60 s (conditioning stimulus, (CS)) on an NRS (0–100). Participants were instructed to spread their fingers and avoid touching the bottom or walls of the water bath. Thirty seconds after the start of the CS, the TS was reapplied to the volar forearm next to the previous site, and participants rated the pain intensity at 10, 20 and 30 s (TS_during_). After completing TS_during_, CS, and a 5‐min break, the TS was repeated without the CS, and participants reported pain intensity at 10, 20 and 30 s (TS_after_).

### 2.4 Conditioned Itch Modulation (CIM)

In order to minimise possible carry‐over effects on results of the subsequent CIM test, we implemented a 1‐h break between CPM and CIM tests. A schematic of the testing procedure can be found in Figure 1d. All participants were tested once. The test site was randomised and to be either the left or right volar forearm. For this study, a voltage generator (DS 5 Digitimer Ltd., Welwyn Garden City, UK), an analog‐to‐digital converter (National Instruments, NIB‐USB 6221, Hungary), a Labview 2018 program running on a laptop computer, a wire electrode (Ø 1 mm, 7 mm length), a disc electrode (Ø 1 cm) (both made at UKM, Münster) and a plastic tub (30 × 20 × 12 cm length × width × height) filled with 10°C water were used. For optimal electrical itch generation, the individual parameters from different previous paradigms (Solinski and Rukwied 2020) were optimised to provide robust and well controlled itch stimuli. Participants were asked to separately rate the perceived itch vs. pain intensity (NRS 0–100) and whether they felt an urge to scratch.

Electrical impulses were applied transcutaneously via the electrode on the volar side of the ipsilateral wrist. We used square‐wave pulses with a frequency of 50 Hz that have been shown to be effective in previous paradigms (Andersen et al. 2017; Edwards et al. 1976; Ikoma et al. 2005; van Laarhoven et al. 2016). The stimulus parameters were adapted to individual participants, such that the itch intensity of a 30 s electrical stimulus was rated at least 30 on an NRS from 0 to 100. For this purpose, the pulse width was increased from 2 to 10 ms (in steps of 2 ms) at a fixed current intensity of 0.12 mA (each test run for 30 s). Only if the itch intensity at maximal pulse width (10 ms) was below 30 (NRS 0–100) was the current intensity increased progressively (to a maximum of 0.24 mA). At these increased current intensities, the pulse width was increased again from 2 to 10 ms in order to minimise the applied charge. For further testing that commenced after a pause of at least 5 min, the individual settings to induce an itch intensity of at least 30 (NRS 0–100) and the urge to scratch the stimulated skin were used. The electrical (TS) was applied for 120 s before, during and after the conditioning painful stimulus (TS_before_, TS_during_, TS_after_, Figure 1d). The perceived intensity of itch and pain, as well as the urge to scratch, was assessed shortly before the start of the stimulation and every 20 s during the stimulations. After a break of 5 min, a CS was applied by immersing the contralateral hand in the water tub (10°C water temperature) up to the level of the wrist 30 s before the start and until the end of the electrical stimulation (Figure 1d). The pain intensity (NRS 0–100) induced by the water bath was assessed every 30 s. After another 5 min pause, the TS was applied again for 120 s, and participants were asked to separately rate their itch and pain sensation as described above.

### 2.5 Skin Biopsies and Measurement of Intraepidermal Nerve Fibre Density

Two skin punch biopsies were obtained according to standard procedures from the same body side that was assessed by QST (for details see (Baka et al. 2024)) located 10 cm above the lateral malleolus (lower leg) using a disposable 6‐mm punch under local anaesthesia. All skin samples were processed to assess intraepidermal nerve fibre density (IENFD) and the presence of inflammatory changes according to a published protocol (Üçeyler et al. 2013). Skin biopsies were analysed at a single centre (Würzburg), with IENFD determined by standardised counting rules by an investigator blinded to subject allocation.

### 2.6 Sample Size Calculation and Data Analysis

Given the exploratory nature of the study and the absence of prior CIM data in PNP patients, no formal a priori sample size calculation was performed. Our group sizes are in line with prior CPM/CIM studies (Andersen et al. 2017; van Laarhoven et al. 2016). Statistical analyses were conducted using IBM SPSS Statistics version 23.0, GraphPad Prism 9 for Windows, and OriginPro 2023. The significance level was set at *p* < 0.05. Group comparisons across the three study groups were conducted using Welch's one‐way ANOVA, which is robust to unequal variances and group sizes. Dunnett's T3 post hoc tests were applied to identify between‐group differences without assuming homogeneity of variances. Because the normality assumption could not be guaranteed, we additionally performed a sensitivity analysis using Kruskal–Wallis tests with Dunn's post hoc tests (Holm correction). Both approaches yielded concordant results. Categorical data were analysed with the *χ* ^2^ test. To evaluate QST parameters without the influence of physical dimensions, a Z‐transformation (mean ± SD, control group) was applied to the QST data. No data imputation was performed for missing data. The mean ± standard deviation (SD) will be used for all values, unless a different representation is indicated.

The immediate (prolonged) CPM effect was calculated by determining the difference between the mean of the three pain ratings for the TS_before_ without the CS and the mean of the three pain ratings for the TS_during_ (5 min after) the CS, relative to the total pain rating for the TS without the CS (immediate CPM effect = (mean of three pain ratings TS_during_—mean of three pain ratings for TS_before_)/TS_before_). The CPM effect was defined as the percentage of the endogenous inhibitory effect, represented by a negative percentage value for the reduction of pain ratings of the TS during CS. Conversely, pain facilitation was shown by a positive percentage value (Yarnitsky et al. 2012).

We analysed the CIM effects as absolute differences and percentage change of NRS between area under the curve (AUC) of ratings during first TS_before_ and TS_during_ simultaneous application of CS (‘immediate CIM effect’) and also between AUC of ratings during first TS and TS 5 min after the application of CS (‘Prolonged CIM effect’). Statistical comparisons were performed not only between baseline (TS_before_) and CS (TS_during_), but also between TS_after_ and both TS_before_ and TS_during_ to assess prolonged CIM effects.

## 3 Results

### 3.1 Demographics and Clinical Characteristics

A total of 48 participants were recruited. However, nine of 48 participants (18.8%) were excluded because they did not reach the predefined threshold of electrically induced itch (≥ 30 NRS). Importantly, exclusions were distributed across groups and not confined to patients with pruritus: two of 18 PNP patients with pruritus (11.1%), five of 13 PNP patients without pruritus (38.5%) and two of 17 controls (11.8%). The final study population therefore consisted of 39 participants (25♀/14♂). The median age of participants was 57 years (range 27–81), with a median disease duration of 72 months (range 10–400). Participants were divided into three groups: PNP with pruritus (PNP_PRU_, *n* = 16), PNP without pruritus (PNP_NO‐PRU_, *n* = 8) and control group (CONTROL, *n* = 15). The median age of CONTROL is 56 years (range 48–59), for PNP_PRU_ is 55.5 years (range 29–81) and for the PNP_NO‐PRU_ group is 43 years (range 27–62) (Figure 1a and Table 1).

Common diagnoses included small fibre Neuropathy (SFN, *n* = 29) and sensorimotor polyneuropathies (*n* = 10); most common (co‐)morbidities were hypertension (aHT, *n* = 15), diabetes mellitus type 2 (DM Typ2, *n* = 5), asthma (*n* = 5) and hypothyroidism (*n* = 10). Other notable comorbidities include depression (*n* = 6), psoriasis (*n* = 3), Hashimoto's thyroiditis (*n* = 4) and various allergies (*n* = 8).

A variety of medications were taken by patients, including gabapentin (*n* = 5), amitriptyline (*n* = 2), ketamine (*n* = 2) and cannabis‐based medicinal extracts (*n* = 3) for pain management; olmesartan (*n* = 1), ramipril (*n* = 4), bisoprolol (*n* = 3) and lisinopril (*n* = 2) for hypertension management; L‐thyroxine (*n* = 6) for thyroid hormone replacement; metformin (*n* = 3) and sitagliptin (*n* = 2) as anti‐diabetics; and other medications, including atorvastatin (*n* = 5), hydrocortisone (*n* = 1), mometason (*n* = 1) and omeprazole (*n* = 2). A concise overview of demographics and comorbidities is provided in Table 1.

### 3.2 Sensory Profiles Indicate Aδ and Aβ Fibre Dysfunction in PNP Patients With Pruritus

**FIGURE 2:**
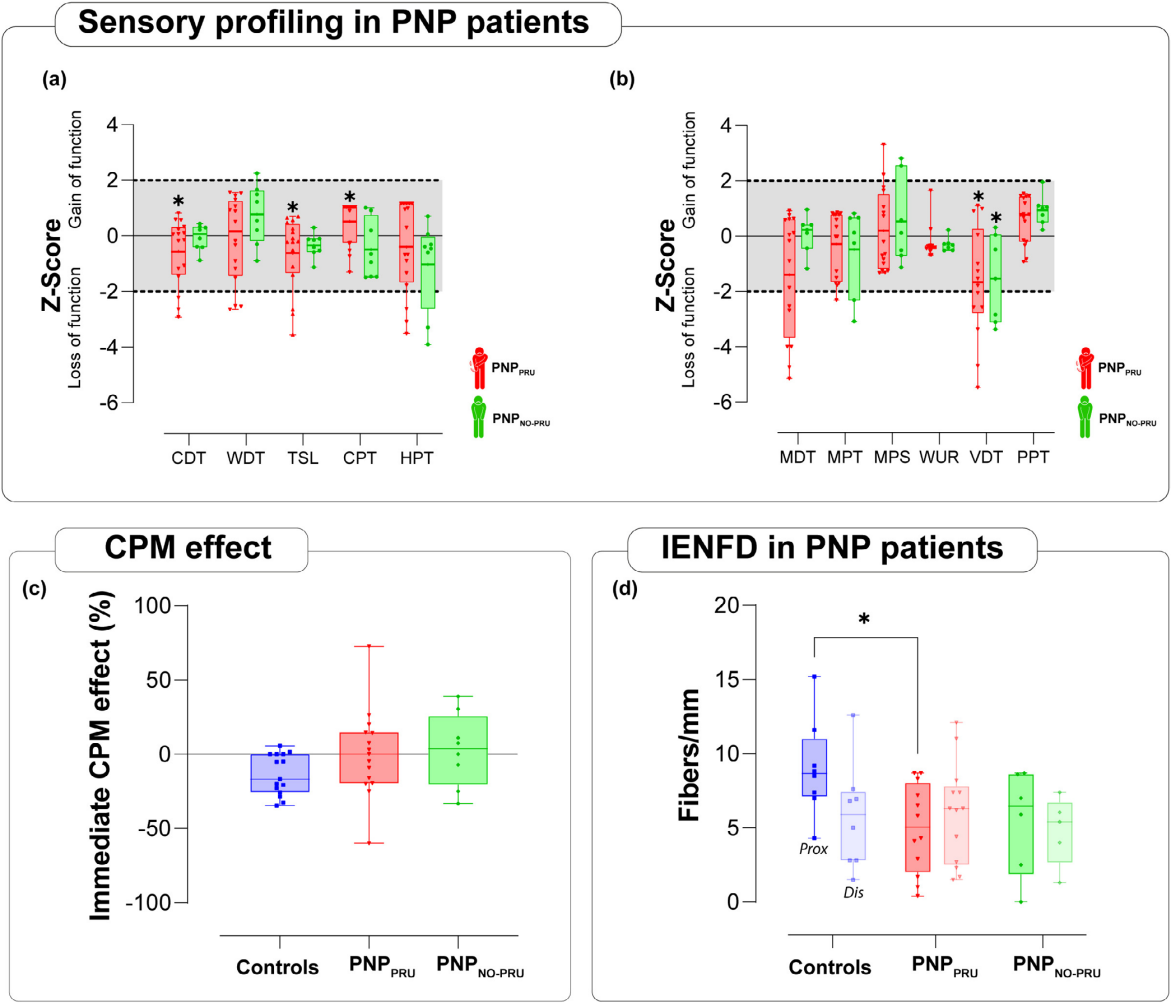
Sensory deficits, impaired CPM, and reduced intraepidermal nerve fibre density in PNP_PRU_ patients. (a) Thermal profiling: PNP_PRU_ patients showed a significant increase in Cold Detection Threshold (CDT) (*p* < 0.05) and altered TSL vs. healthy controls (*p* < 0.05). (b) Mechanical profiling: Both patient groups exhibited a significant increase in Vibration Detection Threshold (VDT) (*p* < 0.05). QST metrics are presented as *Z*‐scores, calculated by using our control group as reference. Alongside the mean (indicated by a line) and the interquartile range (shown as whiskers), the individual scores are presented. Scores above 0 show a gain in function versus controls, while scores below 0 indicate a loss of function versus controls. (c) In the control group, the immediate CPM effect was −13.62% (±SD 13.76), while in PNP_PRU_ patients it was 0.3% (±26.56) and in PNP_NO‐PRU_ patients it was 2.8% (±24.9). Independent samples, no significance. (d) PNP_PRU_ exhibited a significantly diminished proximal IENFD compared to both the PNP_NO‐PRU_ and controls (*p < 0.05). Independent samples. Statistical analyses: Group comparisons across the three study groups were conducted using Welch's one‐way ANOVA (Brown–Forsythe) with Dunnett's T3 post hoc tests. As the normality assumption could not be guaranteed, we additionally performed a Kruskal–Wallis sensitivity analysis with Dunn's post hoc tests (Holm correction), which confirmed the results. PNP_PRU_ (*n* = 16, red), PNP_NO‐PRU_ (*n* = 8, green), controls (*n* = 15, blue) for QST and CPM. PNP_PRU_ (*n* = 12 for proximal, *n* = 13 for distal, red), PNP_NO‐PRU_ (*n* = 6 for proximal, *n* = 5 for distal, green), controls (*n* = 8 for proximal, *n* = 8 for distal, blue) for IENFD analysis. CDT, cold detection threshold; CPT, cold pain threshold; HPT, heat pain threshold; MDT, mechanical detection threshold; MPS, mechanical pain sensitivity; MPT, mechanical pain threshold; PPT, pressure pain threshold; TSL, thermal sensory limen; VDT, vibration detection threshold; WDT, warmth detection threshold; WUR, wind‐up ratio.

PNP_PRU_ patients exhibited a significant elevation in the Cold Detection Threshold (CDT) (*p* < 0.05) compared to healthy controls, showing lowered sensitivity to cold, but were hypersensitive to painful cold (Figure 2a and Table 2). In addition, Thermal Sensory Limen (TSL) was significantly reduced in PNP_PRU_ patients, suggesting impaired thermal discrimination. The Warm Detection Threshold (WDT) and Heat Pain Threshold (HPT) did not differ between the PNP patient groups. The combination of reduced cold sensitivity and impaired thermal discrimination indicates a functional deficit in Aδ fibre processing and impaired integration of Aδ and C fibre input in PNP_PRU_ patients. Both patient groups showed a significant increase in the Vibration Detection Threshold (VDT) (*p* < 0.05), indicating reduced sensitivity to vibratory stimuli. In PNP_PRU_ patients, the Mechanical Detection Threshold (MDT) was elevated, showing a trend towards decreased mechanical sensitivity, though this did not reach statistical significance (Figure 2b). This finding suggests mechanical hypaesthesia and impairment in the Aß‐fibre network in both groups of PNP patients.

**TABLE 2:** Quantitative sensory testing raw data in the original physical dimension.

	Healthy controls	PNP_PRU_	PNP_NO‐PRU_
CDT [°C]	−4.68 (4.49)	**−7.21 (5.38)**	−4.95 (2.14)
WDT [°C]	9.54 (3.21)	10.05 (4.94)	7.05 (3.62)
TSL [°C]	14.50 (8.73)	**19.88 (11.60)**	11.53 (3.95)
PHS [absolute]	1.00 (1.11)	0.75 (1.24)	1.25 (1.29)
CPT [°C]	11.39 (10.39)	**5.73 (8.19)**	16.71 (11.63)
HPT [°C]	47.29 (2.33)	46.34 (3.88)	44.19 (4.12)
MDT [mN]	11.13 (11.17)	30.21 (29.76)	9.88 (7.72)
MPT [mN]	47.94 (47.50)	117.56 (185.63)	64.37 (74.62)
MPS [NRS]	1.23 (0.92)	5.88 (11.21)	2.18 (2.20)
DMA [NRS]	0.00 (0.00)	0.21 (0.59)	0.10 (0.21)
WUR	4.90 (7.23)	3.38 (4.10)	2.68 (1.83)
VDT [/8]	7.30 (0.63)	**5.47 (2.45)**	**6.35 (0.99)**
PPT [kPa]	621.64 (258.60)	466.25 (217.31)	364.88 (142.20)

*Note:* Values are presented as mean ± SD. Bold font indicates a *p*‐value < 0.05 vs. healthy controls when compared with controls.

Abbreviations: CDT, cold detection threshold; CPT, cold pain threshold; DMA, dynamic mechanical allodynia; HPT, heat pain threshold; MDT, mechanical detection threshold; MPS, mechanical pain sensitivity; MPT, mechanical pain threshold; PHS, paradoxical heat sensation; PPT, pressure pain threshold; TSL, thermal sensory limen; VDT, vibration detection threshold; WDT, warmth detection threshold; WUR, wind‐up ratio.

### 3.3 Trends Towards Impaired CPM in PNP Patients

The immediate CPM effect was −13.6% (±13.8) in controls, 0.3% (±26.6) in PNP_PRU_ patients and 2.8% (±24.9) in PNP_NO‐PRU_ patients (Figure 2c). The TS temperature was similar across the groups, showing no significant variation (Figure S1a). Although CPM values were numerically lower in both PNP groups (Table S1), no significant differences were detected across groups (*p* > 0.05).

### 3.4 Reduced Proximal Intraepidermal Nerve Fibre Density in PNP Patients With Pruritus

IENFD was measured at proximal and distal sites in skin biopsies from all 48 participants, including those excluded from CIM due to insufficient itch response (Figure 2c). The mean IENFD at proximal sites was as follows: PNP_PRU_: 4.48 (±3.12); PNP_NO‐PRU_: 5.04 (±3.62) and controls: 8.17 (±3.55). At distal sites, the mean IENFD was 5.85 (±3.38) for PNP_PRU_ 4.52 (±2.58) for PNP_NO‐PRU_ and 5.26 (±3.71) for controls. The PNP_PRU_ group showed a significantly reduced IENFD at the proximal biopsy site compared to controls (*p* < 0.05) (Figure 2d).

### 3.5 Comparable Itch Sensation and Stability of Responses in PNP Patients

**FIGURE 3:**
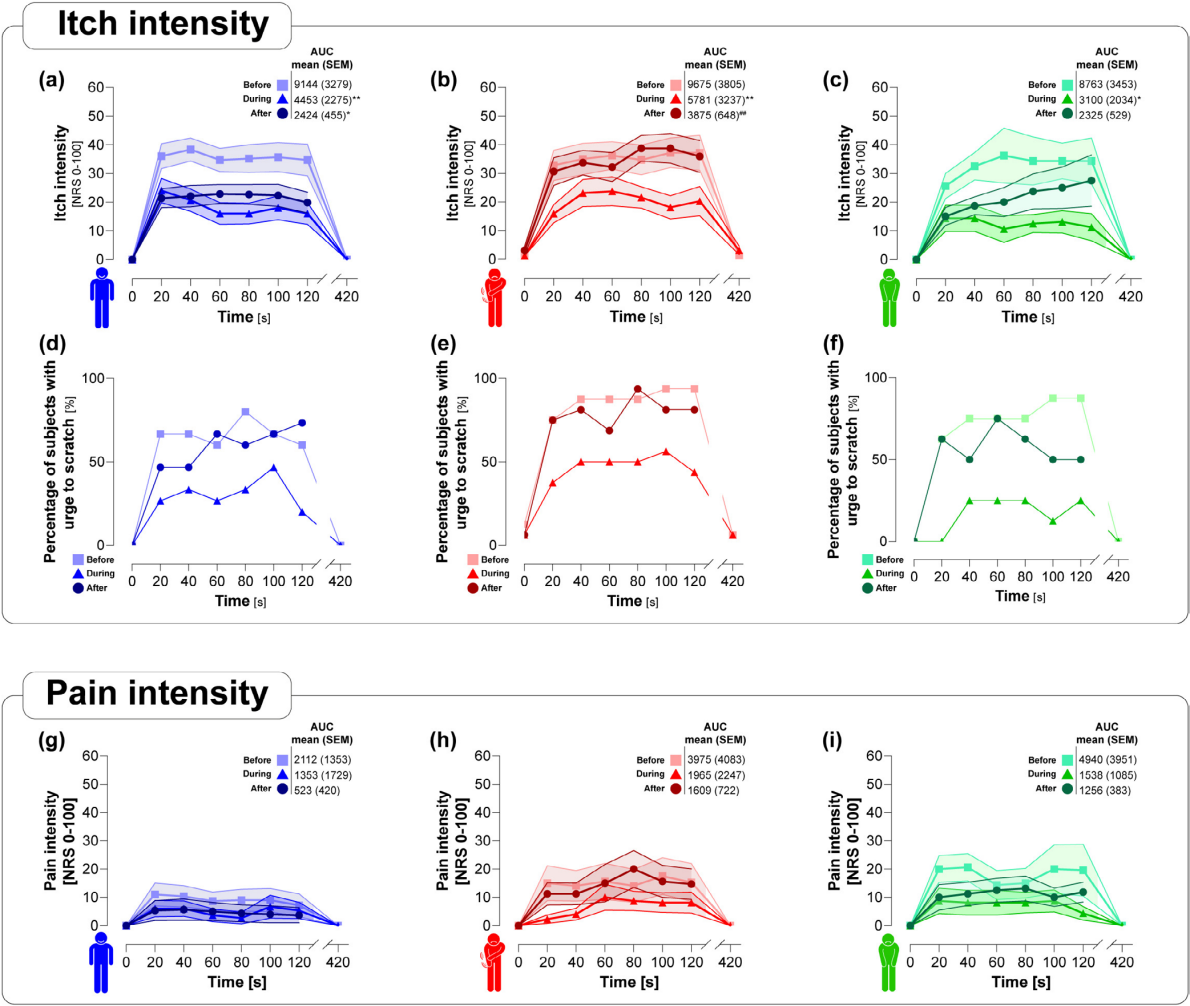
Comparable itch and pain responses across groups during electrical stimulation. (a–c) Line dot plots of absolute change over time for itch intensity, (d–f) urge to scratch and (g–i) pain intensity to the TS before, during and after immersion with CS in controls (blue), PNP_PRU_ (red) and PNP_NO‐PRU_ patients (green). To illustrate the longitudinal trajectories, the mean value is displayed (dot) along with the area indicating SD.

The individualised electrical stimulation induced a similar itch sensation (controls: 26.8 (±21.8); PNP_PRU_: 27.0 (±23.5); PNP_NO‐PRU_: 24.7 (±22.1); Figure 3a–c) as expected. No significant differences in the electrical stimulation parameters were found between the groups (Figure S1b,c). Itch was coupled with the need to scratch in the controls (73.3%), the PNP_PRU_ group (87.5%) and the PNP_NO‐PRU_ group (100%) (Figure 3d–f) and was accompanied by low levels of pain (Figure 3g–i). The sensations were consistent over the application time of 120 s and were completely reversible after a further 300 s. No difference was detected between the groups for pain or itch intensity or the temporal profiles. Although the urge to scratch commonly accompanies itch, it is not strictly required for its definition. Here, the urge was reported in most but not all participants, consistent with individual variability in pruriceptive processing.

### 3.6 Impaired Persistence of Conditioned Itch Modulation in PNP Patients With Pruritus

**FIGURE 4:**
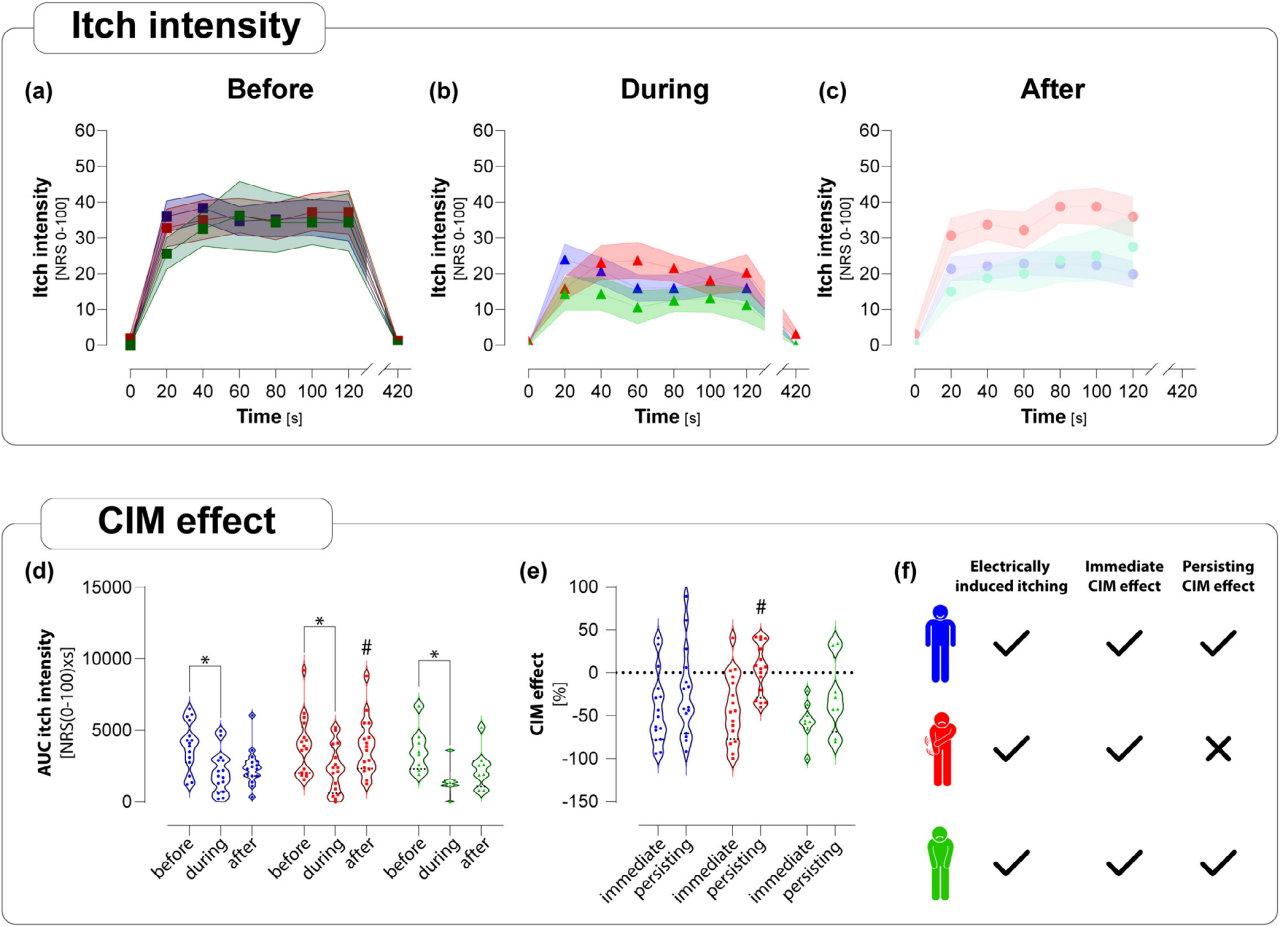
Altered conditioned itch modulation in PNP_PRU_ patients despite similar immediate CIM effects. (a–c) Line plots of the absolute itch intensity before, during and after immersion with CS in controls (blue), PNP_PRU_ (red) and PNP_NO‐PRU_ (green). To illustrate the longitudinal trajectories, the mean value is displayed (dot) along with the SD (area). (d) Bean plots of AUC itch intensity over time before, during and after application of CS for controls (blue), PNP_PRU_ (red) and PNP_NO‐PRU_ (green). Median ± 95% CI *p < 0.05 compared to before stimulus rating. Each dot represents response of one patient. ^#^ *p* < 0.05 compared to during the stimulus rating. (e) Bean plots of relative changes of itch intensity for controls (blue), PNP_PRU_ (red) and PNP_NO‐PRU_ (green). Median ± 95% CI. Immediate CIM effect being the relative change from CS_before_ to CS_during_ and persisting being the relative change from CS_before_ to CS_after_. ^#^ *p* < 0.05 compared to CIM‐effect of controls (f) Schematic summary and comparison of aforementioned results for controls (blue), PNP_PRU_ (red) and PN‐no‐itch patients (green). PNP_PRU_ (*n* = 16, red), PNP_NO‐PRU_ (*n* = 8, green), controls (*n* = 15, blue).

Ratings of electrically‐evoked itch intensities (NRS 0–100) before the CS showed no significant differences in mean AUC between the groups (controls: 9144 (±3279); PNP_PRU_: 9675 (±3805); PNP_NO‐PRU_: 8763 (±3453); Figure 4a–c). Within each group, the itch intensities during the painful CS were significantly lower compared to the baseline levels (controls: 2053.3, *p* < 0.05; PNP_PRU_: 2265.6, *p* < 0.05; PNP_NO‐PRU_: 1412.5, *p* < 0.05; Figure 4d). This reduction was observed in each group as an immediate CIM effect (controls: −41.7 (±11%); PNP_PRU_: −41.5 (±10%); PNP_NO‐PRU_: −55.7 (±8.1%); Figure 4e). PNP_PRU_ showed significantly higher itch intensities in response to the TS after the CS (PNP_PRU_: 3.6% ± 7.3%, *p* < 0.05) compared to both controls and PNP_NO‐PRU_ (controls: −23.3 (±13.4%); PNP_NO‐PRU_: −28.5 (±15.3%)) (Figure 4c,e). Controls and PNP_NO‐PRU_ exhibited a persisting reduction of itch while PNP_PRU_ did not (Figure 4e,f). Within‐group comparisons revealed significant itch reduction during CS (TS_during_ vs. TS_before_) in all groups. Only in the PNP_PRU_ group did itch return to baseline after the CS (TS_after_ vs. TS_before_: ns), while controls and PNP_NO‐PRU_ showed continued suppression (TS_after_ vs. TS_before_ and TS_after_ vs. TS_during_: both *p* < 0.05).

## 4 Discussion and Conclusions

In the current study, we investigated descending inhibition of pain and itch that is induced by painful stimulation of a distant body region and critically involves supra‐spinal processing. CPM was—although not significantly different between groups—almost absent in patients with PNP, regardless of whether pruritus was a PNP symptom or not. No significant differences were found in the immediate CPM and CIM effects among the groups. However, CIM lasted significantly shorter in patients with pruritus, suggesting a decrease in endogenous itch inhibition in this group of patients.

The descending inhibition has been described as an important neuronal pathway in pain processing and modulation (Ikoma et al. 2006). A reduced descending inhibition of pain has been shown by using CPM in chronic pain patients and is considered important in pain chronification (Lewis et al. 2012). In patients with chronic pruritus, reduced CPM has been shown as well, showing that descending inhibition is somehow impaired (Edwards et al. 2003; Pogatzki‐Zahn et al. 2020; van Laarhoven et al. 2010). However, the role of descending inhibition for pruritus in patients is still unclear. In healthy volunteers, descending inhibition evoked by painful stimuli has been shown to inhibit experimental itch and pain (Andersen et al. 2017). Similarly, electrically induced itch was inhibited by histamine‐induced itch in controls, but increased in chronic itch patients, whereas electrically induced pain was inhibited by cold pain in controls, but unchanged in psoriasis patients with chronic itch (van Laarhoven et al. 2010). However, the induction of CIM, apart from CPM, remains a topic of debate, particularly whether a specific itch inhibitory pathway is relevant (Andersen et al. 2017; van Laarhoven et al. 2010). Here, we observed no significant group differences in CPM responses. Both PNP groups showed only minimal CPM effects indicating a reduced endogenous inhibitory capacity for pain in individuals with PNP.

In contrast to CPM, CIM results differed between the groups. To investigate descending inhibition of itch, we made use of an optimised electrical itch paradigm that provided reliable and well‐controlled itch suitable for the assessment of CIM (Ikoma et al. 2005). Our results, showing a decreased inhibition of itch in PNP_PRU_, demonstrate for the first time an impaired endogenous itch inhibition in patients with neuropathic pruritus, and here especially in those patients with pruritus. It remains unclear whether the impaired inhibition is the result of the chronic pruritus or the prerequisite for developing chronic pruritus or both. Because the pain phenotype was similar in both PNP groups, the difference in itch inhibition cannot be a result of pain supporting a separate itch descending pathway.

Our findings of altered endogenous itch inhibition in PNP patients with pruritus suggest a potential pathway for developing targeted therapies to enhance itch control, particularly by supporting endogenous inhibitory mechanisms. Analogous to findings in painful diabetic neuropathy—where patients with reduced CPM show greater benefit from duloxetine because of its modulation of serotonin and norepinephrine systems (Yarnitsky et al. 2012)—patients with chronic pruritus and impaired itch inhibition might respond better to treatments targeting these pathways. Additionally, since a pronociceptive modulation pattern has been associated with pain chronification (Edwards et al. 2003; Yarnitsky et al. 2008), evaluating endogenous itch inhibition in chronic pruritus patients could provide insights into the risk of itch persistence. Reduced IENFD and decreased CPM responses may reflect a predisposition to chronic pruritus, similar to markers that predict chronic postsurgical pain. Carefully directed treatments to bolster these inhibitory processes may help mitigate or prevent itch chronification in patients with these indicators.

### 4.1 Establishing a Reliable Electrical Paradigm to Induce Itch Sensation

We used an electrical, itch‐inducing stimulus as TS in our CIM paradigm. There have been different approaches to generate a reliable itch sensation by electrical stimulation (Andersen et al. 2017; Solinski and Rukwied 2020; van Laarhoven et al. 2010). For our study, we used the protocol by Ikoma and colleagues and varied distinct electrical parameters to induce a reliable pure itch sensation (Ikoma et al. 2005). Instead of a variable stimulation frequency, as chosen by previous CIM paradigms (Andersen et al. 2017; Edwards 1976; van Laarhoven et al. 2007), we chosen a consistent frequency of 50 Hz. It is presumed that C‐ and A‐fibres are stimulated by the used high frequency stimulation (Solinski and Rukwied 2020). The range of pulse duration was slightly different (2–10 ms instead of 0.08–8 ms (Ikoma et al. 2005)), the maximal intensity was doubled to 0.24 mA and the duration of stimulation was 120 s (Andersen et al. 2017; van Laarhoven et al. 2010). These modulations resulted in a new protocol allowing for generating a reliable itch sensation. By using this paradigm in our study, it was possible to generate a sensation of itch in healthy volunteers (control group) and PNP patients with and without pruritus, which did not show any significant differences in intensity between the groups. An additional important aspect was that this paradigm did only produce little pain sensation.

### 4.2 Distinct Sensory Profiles and Peripheral Nerve Alterations in PNP Patients With Pain Versus Pruritus

Both PNP patient groups exhibited a sensory deficiency for vibratory stimuli, while patients with pruritus were less sensitive to cold stimuli but more sensitive to cold pain. Skin biopsies revealed significantly reduced proximal IENFD in patients with pruritus. As expected, PNP patients had deficits in small fibre function, with thermal detection being impaired earlier in the disease than mechanical pain detection, as shown in longitudinal studies (Brask‐Thomsen et al. 2024; Tsilingiris et al. 2024). Patients with chronic inflammatory itch show a similar loss of of C‐fibres function (increased WDT), along with decreased intraepidermal nerve fibre density and impaired VDT (Pogatzki‐Zahn et al. 2020). Here, our focus was on potential differences between both PNP patients' groups. In line with the full cohort (Baka et al. 2024), pruritus coincided with reduced cold perception and diminished IENFD at the thigh. Although IENFD was not directly correlated with CIM outcomes, the inclusion provides context regarding peripheral structural changes and may inform future mechanistic studies. We found increased sensitivity to cold pain in PNP patients with pruritus, indicating hyperexcitability of very superficial nociceptive sensory endings. In addition to activation of specific cold‐sensitive channels, cold‐sensitive potassium channels are closed in the sensory endings, which increases membrane resistance. Enhanced membrane resistance contributes to the generation of spontaneous activity, a mechanism characterised in cultured human dorsal root ganglion neurons obtained from individuals with painful neuropathy (Tian et al. 2024) and might possibly contribute to cold‐induced increase in electrically induced pain (Pakalniskis et al. 2023) or histamine‐induced itch in volunteers (Pfab et al. 2006).

### 4.3 Limitations

As CPM and CIM paradigms assess changes of pain or itch relative to baseline levels, it is unclear to which extent descending inhibition is already active under baseline conditions, in particular in patient populations. As shown experimentally with capsaicin‐induced pain (Ferreira et al. 2010), pre‐existing pain at baseline already activates descending inhibition and thereby the CS is less effective. Thus, non‐evoked itch in the PNP patients with pruritus might facilitate itch processing and thereby limit the duration of the CIM in our study without indicating a weaker descending inhibition of itch. Importantly, this limitation only refers to the CIM, but not the acute induction of electrical itch, which was not facilitated in the PNP patients with pruritus. Approximately 20% of PNP patients failed to reach the required itch threshold during electrical stimulation, indicating possible peripheral dysfunction.

Another concern is the limited statistical power, attributed to substantial variability within our patient groups. Since there is limited knowledge of the exact nerve fibres and neuronal pathways that are targeted by the electrical stimulation, the exact peripheral mechanisms behind the CIM paradigm are not yet fully understood. Further investigation of the itch processing neuronal system, e.g., the central modulation, is necessary to acquire a more detailed impression of the interrelations between nerve damage due to PNP, the development of chronic itch and a dysfunctional endogenous inhibition.

### 4.4 Conclusion

Using a novel protocol for electrically induced itch in a CIM paradigm, our results suggest that PNP patients with chronic pruritus have a shorter‐lived descending itch inhibition that might contribute to itch severity. It remains to be shown to which extent therapeutic interventions targeting this difference can improve neuropathic itch in these patients.

## Author Contributions

J.E., S.B., D.C.R. and D.S. developed testing protocols under the help of M.S. and performed experiments. J.E. preformed statistical analyses and mainly wrote the manuscript together with D.S. D.S. and E.P.‐Z. wrote the ethical application. J.E., S.B., D.C.R. and D.S. wrote the manuscript together with E.P.‐Z. and M.S., F.B., C.S. D.S. and J.E. created figures and tables. E.P.‐Z. conceived, designed, and supervised the study. All authors reviewed the article.

## Supporting information


**Figure S1:** Determination of the test stimulus for CPM and CIM paradigm.

**Table S1:** Absolute CPM values. CPM‐values of the different test runs (before, during and after application of the CS). Absolute values [NRS 0–100 (SD)] of experienced pain are given as means within the groups after 10, 20 and 30 s of the TS. The overall mean expresses the mean pain of the three individual values of the test run within the group. Immediate CPM‐effect = Overall mean (during)—Overall mean (before). Persisting CPM‐effect = Overall mean (after)—Overall mean (before).
