# Supplementary figures and images for: Reduced Descending Itch Inhibition in Peripheral Neuropathy Patients With Chronic Pruritus
Source: Eur J Pain. 2026 Jan 3;30(1):e70190. doi: 10.1002/ejp.70190 (PMC12764315; doi:10.1002/ejp.70190)

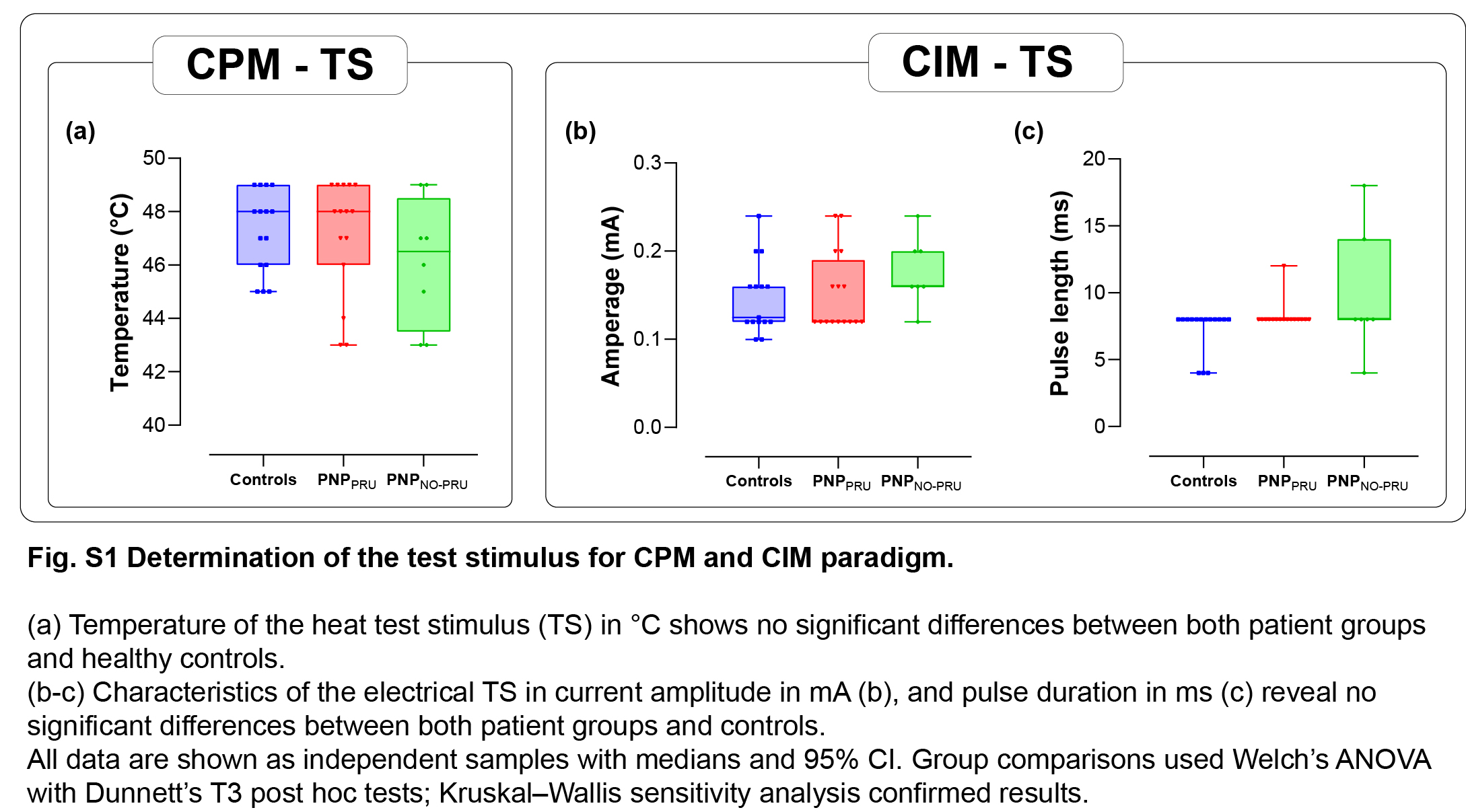

Supplement: Supplementary file 1 — Figure S1: Determination of the test stimulus for CPM and CIM paradigm. [file EJP-30-0-s001.jpg]
