# Supplementary material for: Reduced Descending Itch Inhibition in Peripheral Neuropathy Patients With Chronic Pruritus
Source: Eur J Pain. 2026 Jan 3;30(1):e70190. doi: 10.1002/ejp.70190 (PMC12764315; doi:10.1002/ejp.70190)
Supplement: Supplementary file 2 — Table S1: Absolute CPM values. CPM‐values of the different test runs (before, during and after application of the CS). Absolute values [NRS 0–100 (SD)] of experienced pain are given as means within the groups after 10, 20 and 30 s of the TS. The overall mean expresses the mean pain of the three individual values of the test run within the group. Immediate CPM‐effect = Overall mean (during)—Overall mean (before). Persisting CPM‐effect = Overall mean (after)—Overall mean (before). [file EJP-30-0-s002.docx]

|  | **Before** | | | | **During** | | | | **After** | | | | **Absolute CPM-effect** | |
| --- | --- | --- | --- | --- | --- | --- | --- | --- | --- | --- | --- | --- | --- | --- |
|  | **10s** | **20s** | **30s** | **Overall**  **mean** | **10s** | **20s** | **30s** | **Overall**  **mean** | **10s** | **20s** | **30s** | **Overall**  **mean** | **Immediate** | **Persisting** |
| **Controls** | 52.67  (17.02) | 53.20  (16.45) | 55.13  (18.93) | 53.67  (16.87) | 52.00  (17.49) | 50.00  (19.24) | 51.47  (19.17) | 51.16  (18.21) | 48.00  (16.91) | 45.67  (15.90) | 46.53  (21.16) | 46.73  (16.92) | -2.51  (8.36) | -6.93  (7.09) |
| **PNP_PRU_** | 57.00  (17.11) | 55.53  (17.53) | 54.27  (20.02) | 55.60  (16.84) | 51.33  (20.37) | 46.33  (23.13) | 44.20  (23.39) | 47.29  (21.46) | 59.33  (13.27) | 56.00  (16.75) | 52.67  (21.04) | 56.00  (15.89) | -8.31  (17.98) | 0.40  (18.44) |
| **PNP_NO-PRU_** | 49.38  (10.73) | 43.75  (14.74) | 43.13  (19.03) | 45.42  (14.28) | 39.38  (18.10) | 38.75  (19.49) | 40.00  (21.94) | 39.38  (19.61) | 46.88  (13.68) | 46.25  (16.91) | 45.00  (20.31) | 46.04  (16.26) | -6.04  (14.29) | 0.63  (9.50) |
